# Intracuff Water Droplet Accumulation Occurring Around Eight Hours in Polyurethane-Cuffed Endotracheal Tubes: An In Vitro Simulation Study

**DOI:** 10.7759/cureus.93000

**Published:** 2025-09-23

**Authors:** Naoto Funami, Masafumi Idei, Yohei Sakai, Nobuyuki Yokoyama, Makoto Inoue

**Affiliations:** 1 Department of Critical Care Medicine, Yokohama City University Hospital, Yokohama, JPN

**Keywords:** airway management, airway sealing, critical care, cuff leak, endotracheal tube, intensive care unit, intracuff pressure, mechanical ventilation, ventilator-associated complications

## Abstract

Introduction

Ventilator-associated pneumonia (VAP) is a major complication in intensive care unit (ICU) patients, often resulting from microaspiration due to poor cuff sealing. Polyurethane (PU)-cuffed tracheal tubes, with thinner walls than conventional polyvinyl chloride (PVC) cuffs, offer improved sealing. However, their high water permeability can lead to intracuff water droplet accumulation, potentially interfering with accurate cuff pressure monitoring. This study aimed to determine the timing of water droplet formation in PU cuffs under simulated ventilatory conditions.

Methods

An in vitro simulation was conducted using six tracheal tubes (two each of PU-cuffed Microcuff™ (Kimberly-Clark Health Care, Roswell, GA), PU-cuffed Parker Flex-Tip™ (Parker Medical, Highlands Ranch, CO), and PVC-cuffed TaperGuard™ (Covidien (Medtronic), Mansfield, MA)). Tubes were exposed to heated, humidified airflow for 24 hours at a mean temperature of 39.8 ± 0.4°C and 88 ± 4.6% humidity. Cuff pressure was maintained at 25 cmH₂O. Hourly inspections for visible intracuff water droplets were performed.

Results

Visible water droplets first appeared at eight hours in all PU-cuffed tubes, with a mean onset of 8.25 hours. Fluid migration into the inflation line and pilot balloon was also observed. No water accumulation was seen in the PVC-cuffed tubes. No structural cuff damage was noted.

Conclusion

Water droplet accumulation in PU-cuffed tracheal tubes occurs around eight hours after exposure to humidified ventilation. This early condensation may impair cuff pressure accuracy, posing a safety risk in ICU settings. Clinicians should consider this when managing airway devices and cuff pressures in patients with PU-cuffed tubes.

## Introduction

Ventilator-associated pneumonia (VAP) remains a major complication in intensive care units (ICUs), significantly increasing patient mortality, healthcare costs, and ICU length of stay [1]. One of the key mechanisms behind VAP is the microaspiration of oropharyngeal secretions into the lower airways through folds formed between the tracheal tube cuff and the tracheal wall. Traditional polyvinyl chloride (PVC) cuffs are prone to fold formation during inflation, allowing secretions to leak through [1,2].

To address this issue, thin-walled polyurethane (PU) cuffs were developed. PU cuffs, with a thickness of approximately 10 μm - much thinner than PVC cuffs (50-70 μm) - are highly flexible and conform closely to the tracheal wall, enabling effective sealing at lower cuff pressures. Several in vitro studies have shown that PU cuffs significantly reduce the risk of microaspiration, a major cause of VAP [3-6]. A systematic review by Blot et al. further confirmed the effectiveness of PU cuffs in reducing VAP, especially in postoperative patients expected to require mechanical ventilation [7].

However, the thinness of PU cuffs contributes to their higher permeability to water molecules compared to PVC, potentially leading to water droplet accumulation within the cuff. These droplets can migrate into the inflation line and pilot balloon, interfering with accurate cuff pressure adjustment and monitoring [8,9].

Spapen et al. reported that water droplet accumulation in the PU cuff and inflation line can result in significant underestimation of actual cuff pressure, increasing the risk of both under- and overinflation [8]. In a clinical pilot study, Idei et al. observed visible water droplets within PU cuffs and inflation lines within 24 hours of intubation [9].

Despite these reports, data on the precise timing of water droplet formation within PU cuffs are limited. Previous studies by Spapen et al. and Idei et al. reported the presence of intracuff droplets but did not determine the exact onset time [8,9]. Clarifying this timeline is clinically important, as inaccurate cuff pressure monitoring caused by early condensation could increase the risk of air leakage, VAP, or tracheal mucosal injury.

This study aimed to clarify the timing of water droplet accumulation in PU cuffs using an in vitro model designed to replicate mechanical ventilation conditions with heated and humidified airflow.

A portion of this work was previously presented at the 69th Annual Meeting of the Japanese Society of Anesthesiologists in June 2022.

## Materials and methods

This in vitro simulation study was conducted in the ICU at Yokohama City University Hospital. The tested tracheal tubes included two commonly used PU-cuffed models - Microcuff™ Endotracheal Tube (Kimberly-Clark Health Care, Roswell, GA) and Parker Flex-Tip™ Endotracheal Tube (Parker Medical, Highlands Ranch, CO) - as well as a PVC-cuffed tube, TaperGuard™ Endotracheal Tube (Covidien (Medtronic), Mansfield, MA). All tubes had an internal diameter of 7.5 mm, with two tubes of each type, totaling six tubes.

**Figure 1:**
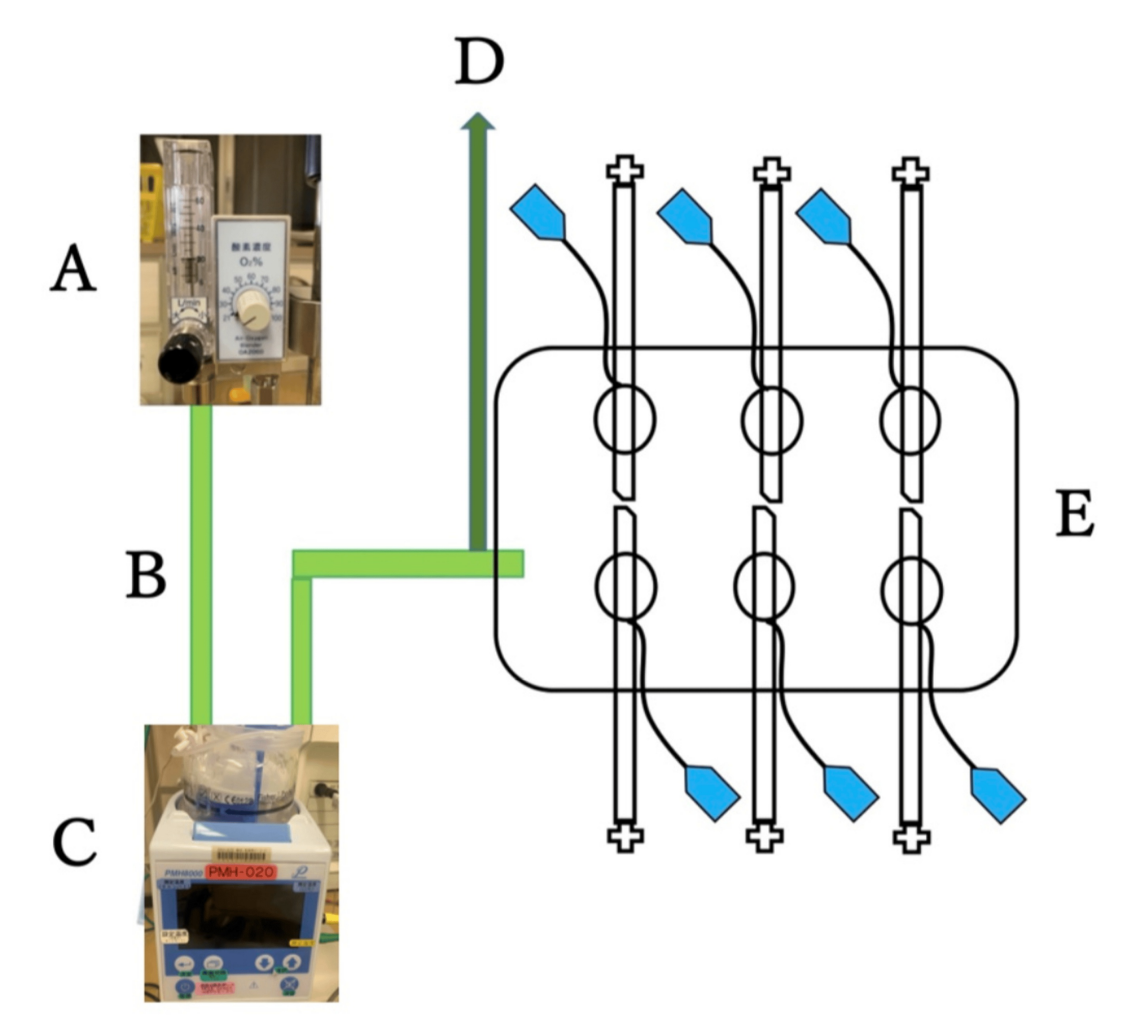
In vitro simulation model replicating a mechanical ventilator circuit with heated and humidified airflow conditions. A: Air-oxygen blender used for high-flow nasal therapy. B: Mechanical ventilator breathing circuit. C: Heated humidifier. D: Temperature and humidity sensor. E: Tracheal tubes inserted into a plastic box. Image credits: Author Naoto Funami

This simulation was carried out in a private ICU room with the ambient temperature carefully regulated at approximately 25°C and humidity maintained at around 40% to ensure environmental consistency. As shown in Figure 1, a simulation environment replicating mechanical ventilation conditions was created inside a plastic box (approximately 20 cm × 9 cm × 7 cm). A mechanical ventilator breathing circuit (Intersurgical Flex Heated Breathing Circuit Sleeve (Adult), Intersurgical Ltd., Wokingham, UK) was connected, and a 20 L/minute flow of air was supplied via an OA2060 Air-Oxygen Blender designed for nasal high flow therapy (Sanei Technology Co., Ltd., Tokyo, Japan). Air was heated and humidified using an MH800 Humidifier (Pacific Medico Co., Ltd., Tokyo, Japan) in auto mode. A sensor was installed in the proximal circuit near the box to measure temperature and humidity hourly. Six ports were drilled into the box to accommodate the six tracheal tubes. Each tracheal tube was randomly assigned to a port, inserted through it, and fixed at the glottic marker position using adhesive tape, ensuring consistent placement across all tubes. Cuff pressure was set to 25 cmH₂O using a Manual Cuff Pressure Gauge (CE0123, VBM Medizintechnik GmbH, Sulz am Neckar, Germany).

Every hour, each cuff was deflated and visually inspected by two anesthesiologists for visible water droplets inside the cuff and inflation line. After each observation, cuff pressure was readjusted to 25 cmH₂O. The observation period lasted up to 24 hours, as guided by a previous study [9]. 

The primary objective of this study was to determine the timing of intracuff water droplet formation in PU-cuffed tracheal tubes under simulated ventilatory conditions. The secondary objective was to explore whether fluid migration could occur as a result of early condensation. To provide further context, we also included a comparison with PVC-cuffed tracheal tubes, which are commonly used in clinical practice, to examine whether condensation is material-dependent.

## Results

During the 24-hour in vitro simulation, the temperature and humidity within the circuit were recorded every hour using in-line sensors, yielding mean values of 39.8 ± 0.4°C and 88 ± 4.6%, respectively.

The initial appearance of visible intracuff water droplets in PU-cuffed tracheal tubes was consistently identified by two independent anesthesiologists. For the Parker™ tubes, both exhibited droplet formation at eight hours. Among the Microcuff™ tubes, one showed droplets at eight hours and at nine hours (Table 1).

**Table 1:** Summary of water droplet formation and migration in tracheal tube cuffs, inflation lines, and pilot balloons. PU: polyurethane, PVC: polyvinyl chloride. “〇” indicates that visible water droplets were observed; “×” indicates none were observed during the 24-hour period. All values are presented as mean values.

Tracheal tube	Cuff material	n	First visible intracuff water droplet (hours)	First visible water droplet migration into inflation line (hours)	Migration into pilot balloon (after 24 hours simulation)	Observed structural damage
Parker™	PU	2	8.0	8.0	〇	None
Microcuff™	PU	2	8.5	8.5	〇	None
TaperGuard™	PVC	2	×	×	×	None

**Figure 2:**
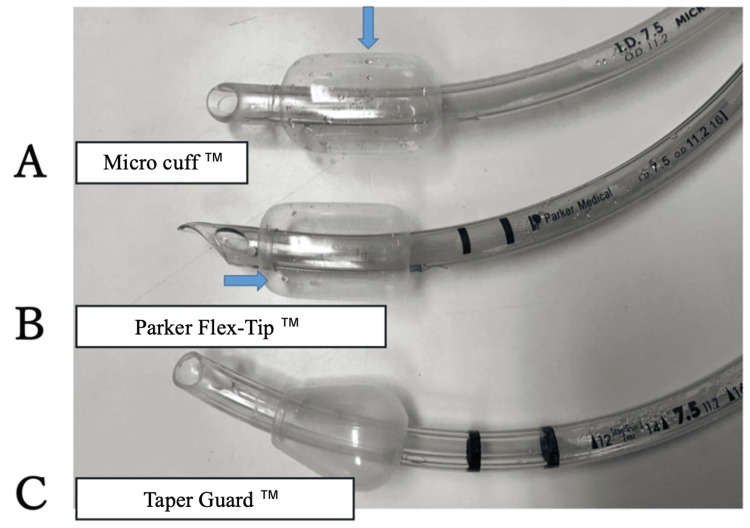
Tracheal tubes after 24 hours of in vitro simulation. A: Microcuff™ Endotracheal Tube (Kimberly-Clark Health Care, Roswell, GA). B: Parker Flex-Tip™ Endotracheal Tube (Parker Medical, Highlands Ranch, CO). C: TaperGuard™ Endotracheal Tube (Covidien (Medtronic), Mansfield, MA). Visible water droplet accumulation was observed in the PU cuffs of both A and B, whereas no droplet formation was noted in the polyvinyl chloride (PVC) cuff of C. Image credits: Author Naoto Funami

**Figure 3:**
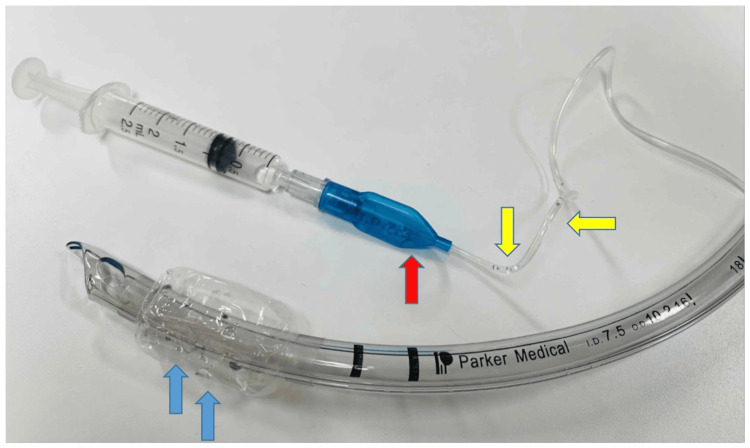
Visible water droplets accumulated within the cuff (blue arrows) of a polyurethane-cuffed Parker Flex-Tip™ Endotracheal Tube (Parker Medical, Highlands Ranch, CO). These droplets can migrate into the inflation line (yellow arrows) and pilot balloon (red arrow), potentially interfering with accurate measurement and adjustment of cuff pressure. Image credits: Author Naoto Funami

Accordingly, the mean onset time of water droplet appearance across all four PU-cuffed tubes was 8.25 hours. At the same time, water droplets were also observed within the inflation lines of the respective tubes. At the conclusion of the 24-hour simulation, all four PU-cuffed tracheal tubes exhibited multiple visible water droplets within both the cuff and the inflation line (Figure 2), and the quantity of fluid was sufficient to migrate into the pilot balloon when negative pressure was applied via a syringe connected to the balloon valve (Figure 3). 

In contrast, neither of the two PVC-cuffed TaperGuard™ tubes exhibited any clearly visible water droplets within the cuff, inflation line, or pilot balloon throughout the entire 24-hour observation period. At the end of the 24-hour simulation, all six tracheal tubes were examined by two anesthesiologists. Visual and manual inspection revealed no structural damage, such as perforation or deformation, in any of the tube cuffs.

## Discussion

This in vitro simulation study demonstrated that water droplet accumulation occurs inside PU-cuffed tracheal tubes within approximately eight hours under conditions simulating heated and humidified ventilation. These findings are consistent with a previous clinical study [9], which reported that visible water droplets had already accumulated in PU cuffs of tracheal tubes that were removed approximately 24 hours after intubation. Our data suggest that such droplets may have begun forming much earlier. The high water permeability of PU is well-documented and likely contributes to this phenomenon [8].

As reported by Spapen et al., a water droplet within the inflation line can increase resistance, causing discrepancies between measured and actual cuff pressures [8]. In ICU settings with intermittent or continuous cuff pressure monitoring, this can lead to either underinflation, risking air leakage and VAP, or overinflation, posing a risk of tracheal mucosal injury [10]. Early intracuff condensation may therefore theoretically contribute to inaccurate cuff pressure monitoring and increase the risk of complications such as VAP; however, these potential links were not directly assessed in our in vitro model, and the translational impact on clinical outcomes should be considered limited. Because significant water accumulation within the cuff may necessitate aspiration of fluid from the pilot balloon or, in some cases, replacement of the tracheal tube, these findings represent an important consideration that should not be overlooked in ventilatory management in the ICU.

Blot et al. showed that PU cuffs are superior to PVC cuffs in preventing microaspiration [7]. Lorente et al. also demonstrated that PU cuffs combined with subglottic secretion drainage significantly reduced early- and late-onset VAP [2]. While these studies support the use of PU cuffs for VAP prevention, our findings highlight the need to consider the potential for inaccurate cuff pressure control due to early condensation. 

In our study, both PU-cuffed tracheal tube models showed similar timing for water droplet formation, indicating that the issue is material-dependent rather than design-specific. Although this study focused primarily on cuff material under standardized ventilatory conditions, other factors such as cuff geometry (tapered vs. cylindrical), cuff volume, and pressure characteristics (high-pressure/low-volume vs. low-pressure/high-volume), as well as tube position, may also influence condensation by altering airflow dynamics and surface contact. These variables were not directly evaluated in this study and should be explored in future investigations. Future studies should investigate other PU-cuffed tracheal tubes and various cuff sizes, including pediatric versions, to confirm these findings.

This study has several limitations. First, the experimental design was in vitro, which, despite careful simulation of ventilatory conditions including temperature, humidity, and cuff pressure, cannot fully replicate the complex physiological environment of the human airway. Key clinical factors such as tracheal wall dynamics, patient movement, and interactions with airway secretions were not reflected in the model.

Second, the sample size was relatively small, as this study was intended as a pilot investigation to explore the feasibility and initial trends related to intracuff condensation in PU-cuffed tracheal tubes. Consequently, caution should be exercised when generalizing these findings to broader clinical settings.

Additionally, the number of PU-cuffed tracheal tube products currently available in Japan is limited, which may restrict the applicability of our results to a wider range of devices used internationally. While observer blinding was not feasible due to the nature of the study, assessments were conducted independently by two observers to enhance objectivity.

Based on these findings, our group is planning a larger-scale clinical study involving ICU patients to further examine the clinical relevance of early intracuff condensation under real-world ventilatory conditions. While our study does not claim superiority of PVC over PU cuffs in clinical outcomes, it emphasizes the importance of evaluating the effects of early intracuff condensation on outcomes such as VAP rates, air leakage, and tracheal mucosal injury. Moreover, our findings may inform future innovation in cuff materials that combine flexibility with resistance to condensation.

## Conclusions

This pilot study demonstrates that, in a simulated mechanical ventilation environment, visible water droplet formation occurs at approximately eight hours in PU cuffs. This early condensation may compromise the accuracy of cuff pressure monitoring and pose a safety risk. Clinicians should be aware of condensation-related pressure management issues from an early stage when using PU-cuffed tracheal tubes in the ICU.
